# Developing the Own-Race Advantage in 4-, 6-, and 9-Month-Old Taiwanese Infants: A Perceptual Learning Perspective

**DOI:** 10.3389/fpsyg.2016.01606

**Published:** 2016-10-19

**Authors:** Sarina Hui-Lin Chien, Jing-Fong Wang, Tsung-Ren Huang

**Affiliations:** ^1^Graduate Institute of Neural and Cognitive Sciences, China Medical UniversityTaichung, Taiwan; ^2^Department of Psychology, National Taiwan UniversityTaipei, Taiwan

**Keywords:** other-race effect, own-race advantage, face processing, perceptual narrowing, perceptual learning, experience, perceptual development

## Abstract

Previous infant studies on the other-race effect have favored the *perceptual narrowing* view, or declined sensitivities to rarely exposed other-race faces. Here we wish to provide an alternative possibility, *perceptual learning*, manifested by improved sensitivity for frequently exposed own-race faces in the first year of life. Using the familiarization/visual-paired comparison paradigm, we presented 4-, 6-, and 9-month-old Taiwanese infants with oval-cropped Taiwanese, Caucasian, Filipino faces, and each with three different manipulations of increasing task difficulty (i.e., change identity, change eyes, and widen eye spacing). An adult experiment was first conducted to verify the task difficulty. Our results showed that, with oval-cropped faces, the 4 month-old infants could only discriminate Taiwanese “change identity” condition and not any others, suggesting an early own-race advantage at 4 months. The 6 month-old infants demonstrated novelty preferences in both Taiwanese and Caucasian “change identity” conditions, and proceeded to the Taiwanese “change eyes” condition. The 9-month-old infants demonstrated novelty preferences in the “change identity” condition of all three ethnic faces. They also passed the Taiwanese “change eyes” condition but could not extend this refined ability of detecting a change in the eyes for the Caucasian or Philippine faces. Taken together, we interpret the pattern of results as evidence supporting *perceptual learning* during the first year: the ability to discriminate own-race faces emerges at 4 months and continues to refine, while the ability to discriminate other-race faces emerges between 6 and 9 months and retains at 9 months. Additionally, the discrepancies in the face stimuli and methods between studies advocating the narrowing view and those supporting the learning view were discussed.

## Introduction

Race is more of a social category than a biological one; the influence of race is implicit but profoundly important (Cosmides et al., 2003). People not only automatically encode the race of each individual they encounter, but they are also better at recognizing faces of their own-race individuals than those of a different race, which is referred to as the own-race advantage (ORA) (Malpass and Kravitz, 1969) or the other-race effect (ORE) (Meissner and Brigham, 2001). During the past 40 years, the ORE has been reliably demonstrated and the effect was robust across different ethnic groups (Carroo, 1986; Valentine and Endo, 1992; Chiroro and Valentine, 1995; Hayward et al., 2008). Although the exact mechanisms remain debatable, it is generally agreed that the ORE stems from a lack of visual experience in processing faces of other unfamiliar race classes. International and transracial adoption studies presented evidence that further endorsed this viewpoint: the adoptees exhibited a diminished (de Heering et al., 2010) or even reversed ORE (Sangrigoli et al., 2005) owing to greater exposure to the other-race classes/faces in childhood and early adulthood.

In the last decade, convergent evidence from cross-cultural research pointed to an early onset of the “ORE,” in the first year of life. Studies based on the spontaneous preference paradigm showed that 3-month-old Caucasian infants, but not newborns, exhibited spontaneous looking preference for Caucasian face when paired with other-race faces (Kelly et al., 2005). Likewise, 3-month-old Chinese infants showed looking preferences for Chinese faces (Kelly et al., 2007a). Ethiopian orphan infants who were frequently exposed to both Ethiopian and Israeli adults preferred African and Caucasian faces equally (Bar-Haim et al., 2006). Thus, it appears that infants at 3 months are sensitive to “race” and their looking preferences are influenced by the faces of the dominant race in early environment. Moreover, two recent studies using preferential looking methods further revealed a rather dynamic development of infant’s preference for own- vs. other-race faces from 3 to 9 months; at 9 months, infants’ spontaneous preference tilts toward other-race face instead (Fassbender et al., 2015; Liu et al., 2015).

The asymmetry in exposure to own- vs. other-race faces continues to augment beyond 3 months. How might the specific type of face inputs from the environment shape infants’ abilities to discriminate among faces of own and other races? To date, several studies have favored the *perceptual narrowing view* and suggested that appropriate visual experience seems necessary for maintaining the neural representations of the faces of certain race classes (Scott et al., 2007; cf. Flom, 2014; cf. Maurer and Werker, 2014). Using the habituation method, Kelly et al. (2007b) tested 3-, 6-, and 9-month-old British Caucasian infants with faces of four ethnic groups, Caucasian, Chinese, African, and Middle Eastern. The infant’s novelty preferences after habituation were taken as an index of successful discrimination between the habituated and the novel face, both presented at a slightly different orientation. The results showed that 3-month-old infants were able to discriminate among faces of all four ethnic groups. 6-month-old infants’ exhibited some form of the ORE; they could only discriminate Caucasian and Chinese faces but not the African and Middle Eastern faces. Lastly, 9-month-old infants could only recognize the own-race Caucasian faces and not any others. With almost identical experimental design, Chinese infants of similar ages were tested with faces of three ethnic groups (African, Chinese, and Caucasian) and the same pattern of results was nicely replicated (Kelly et al., 2009). Three-month-old Chinese infants demonstrated recognition in all three race conditions, 6-month-olds in two conditions (Chinese and Caucasian), and 9-month-olds’ recognition was limited to only Chinese faces. In another study, Ferguson et al. (2009) tested 4- and 8- month-old American Caucasian infants’ ability to assess holistic (i.e., attending to the relationship between both internal and external features) vs. featural (i.e., attending to individual features of the face) information. The results showed that 8-month-olds demonstrated holistic processing of upright own-race (Caucasian) faces, but featural processing of upright other-race (African) faces. However, 4-month-olds demonstrated holistic processing of both Caucasian and African upright faces, suggesting that infants’ processing of own- vs. other-race faces becomes specialized between 4 and 8 months. Across the three studies, it appears that infant’s visual representation of faces is broadly tuned initially; however, continued experience with own-race faces and lack of experience with other-race faces leads to perceptual narrowing, a process in which the sensitivity for unfamiliar other-race faces gradually diminished (Slater et al., 2010; Anzures et al., 2013).

While the literature on perceptual narrowing emphasizes declined sensitivities to rarely exposed stimuli (Nelson, 2001; Scott et al., 2007), research of perceptual learning highlights improved or better sensitivities for frequently exposed stimuli (Sagi, 2011). In contrast to the narrowing view, several studies reported that infants exhibited a readily better discriminability for own-race faces as early as 3 or 4 months. Sangrigoli and de Schonen (2004) was the first to demonstrate ORA at 3 months. After habituated to a Caucasian female face, 3-month-old French Caucasian infants looked longer at a novel Caucasian female face against a habituated one; but they failed to show a novelty preference in the Asian face condition. Using morphing face stimuli, Hayden et al. (2007) reported that 3-month-old American Caucasian infants were better able to differentiate between small differences in facial identity with morphed Caucasian faces; but they were unable to differentiate the same amount of differences among morphed Asian faces. In another recent study, Tham et al. (2015) investigated the relationship between ORE and face gender in 3- to 4- and 8- to 9-month-old British Caucasian infants. They found that, at 3–4 months, infants’ discrimination of Chinese, Malay, and Caucasian faces exhibited ORA for female faces alone, whereas at 8–9 months, the ORA became general across gender.

In addition to the aforementioned studies with Caucasian infants, a recent study with Asian infants showed evidence supporting the perceptual learning view. Using the familiarization/visual paired comparison procedure with oval-cropped faces, Hsu and Chien (2011) tested 4-, 6-, and 9-month-old Taiwanese infants with a set of old/new face discrimination tasks and each comprising three manipulations in the test stimuli (i.e., identity change, change eyes and move up mouth, move up mouth only). The results showed that 4-month-olds can only pass the Asian “change identity” condition, the easiest condition where the two faces were two distinct persons. Six-month-old infants made some progress; they showed novelty preferences in the “identity change” conditions of all three races. Nine-month-old infants not only maintained the recognition for the “identity change” conditions of all three races, but also progressed to the Asian “change eyes and move up mouth” condition, a more difficult condition where the target face is a digitally altered version (i.e., the eyes were replaced with a novel pair of eyes, and the mouth was moved up about 10 pixels closer to the nose) of the original one, indicating a refinement in the ability to process own-race face. Taken together, these findings collectively demonstrated early ORA at about 3 or 4 months, at least for female faces. In addition, Hsu and Chien (2011) further revealed that the continued experience with own-race faces may help facilitate infant’s ability to process finer facial features as they grow (also see Chien and Hsu, 2012).

To summarize the above literature, it seemed that studies favoring the narrowing view and those supporting the learning view are equally abundant. Recently, Vogel et al. (2012) suggested the possibility that early in the first year of life infants (<5 months) may use an attention-based system to discriminate faces, and later in development the same processing shifts to a perceptual-based system that is influenced by previous experiences. Moreover, we know that infants’ retinae are very immature at birth; their spatial vision (i.e., grating acuity and stereopsis) and spectral vision (i.e., color vision) develop substantially during the first year of life (Atkinson, 1983; Simons, 1993; Teller, 1997). It is of interest to ask whether the maturation in the visual capacity helps infants to sharpen their ability to process faces in general, combing the specific effect of own-race face exposure from birth onward. In other words, we hope to explore whether the rate of development differs for processing own- vs. other-race faces, given the asymmetry in the race-relevant inputs from early visual environment.

Thus, the overarching goal of the present study was to investigate whether infants exhibit differential rate of improvement for processing own- vs. other-race faces in the first year, when the visual system undergoes a period of rapid development. Specifically, we asked whether infants’ ability to process own-race faces improves between 4 and 9 months, and whether their ability to process other-race faces also improves between 4 and 9 months, but with a slower rate. This would in turn help us gain insight about the role of continued exposure to own-race faces, a *maintenance* or a *facilitative* type of experience for later development (Gottlieb, 1976; Scott et al., 2007; Flom, 2014; Maurer and Werker, 2014). If maintenance type is true, infants may “grow out of” the capacity to discriminate foreign faces. If the facilitation mode rings true, we predict that with age, infants will show refined capacity in discriminating among own-race faces, while for other-race faces, the ability should be retained as well, just not as refined.

To assess whether infants show improvement in face processing, our approach is to create a comprehensive set of old/new face discrimination tasks with ascending levels of task difficulty for each race. To do so, we modified Hsu and Chien (2011)’s design by making either featural or configural manipulations and created four levels of task difficulty: (1) change identity, (2) change eyes only, (3) widen eye spacing, and (4) move mouth up conditions. These manipulations were chosen based on a body of infant eye tracking studies. Several studies with Caucasian infants had documented that, on a human face, the eyes are typically fixated the most then followed by the mouth area (Maurer and Salapatek, 1976; Haith et al., 1977; Hunnius and Geuze, 2004; Oakes and Ellis, 2013) and that the early face processing seems to shift from featural to configural process (i.e., Mondloch et al., 2002; Schwarzer et al., 2007). A recent eye-tracking study with 4- to 9-month old Chinese infants revealed an interesting observation; with increased age, infants’ fixation time for the nose of other-race faces was decreased but maintained for the nose of own-race faces (Liu et al., 2010). Given the evidence above, we would expect that the “change identity” condition would be the easiest one, followed by the “change eyes” condition (a featural change), and the “widen eye spacing” condition (a configural changes in the eyes region). The “move mouth up” condition shall be the hardest one (a configural change near the mouth area). Additionally, we intend to empirically validate this predicted order of task difficulty with adults’ performance as well. Therefore, an adult experiment was first conducted to ensure appropriate stimulus manipulations and hence a subset of the stimuli can be appropriately applied to infant participants. Thus, the Adult Experiment involved four ethnic groups and each with four manipulations, while the Infant Experiment involved three ethnic groups and each with three manipulations.

## Materials and Methods

### Experiment 1: Adult Study

#### Participants

A total of 22 adult participants (10 males) joined the study (mean age = 22.2). All of participants were naïve to the purposes of the experiment, had normal or corrected-to-normal vision (20/20), and had no close contact with other-race foreigners by self-report. Informed written consent was obtained prior to the experiment. Each participant was tested individually in a quiet room, and received either cash compensation or a course credit for their participation. The experiment protocol adhered to the humanitarian concerns proposed in the Declaration of Helsinki, and was approved by the Central Regional Research Ethics Center, CRREC, Taichung, Taiwan.

#### Apparatus and Stimuli

A desktop computer (Acer Veriton M460) with 22″ LCD monitor (Chimei CMV 221) and E-Prime Professional 2.0 (Psychological Software Tools, Sharpsburg, PA, USA) were used to run the experiment. The stimuli were full-color photos of male and female faces of four ethnic groups, Asian, Caucasian, African, and Filipino. The Asian faces were selected from the Taiwanese Facial Expression Image Database, TFEID (Chen and Yen, 2007), while the Caucasian and African faces were from the NimStim Face Stimulus Set (Tottenham et al., 2009). Female Filipino face photos were migrant workers in Taiwan taken by us with permission in Taichung. The skin tones of individual faces within the same race were equated using PhotoImpact 10 (Ulead System, Taipei) so as to reduce differences in color and luminance. In addition, to remove the background and external cues such as hair, hairline, and ear, all face images were oval-cropped, resized to the same height and width, and mounted on a black background. The size of the oval-shaped faces extended about 11.5 cm (width) by 13.5 cm (height) on the LCD monitor, which was equivalent to about 13.8 by 16.2° of visual angle, with a viewing distance of approximately 50 cm. The monitor was framed by black cardboard to match the black background of the stimuli display.

**FIGURE 1:**
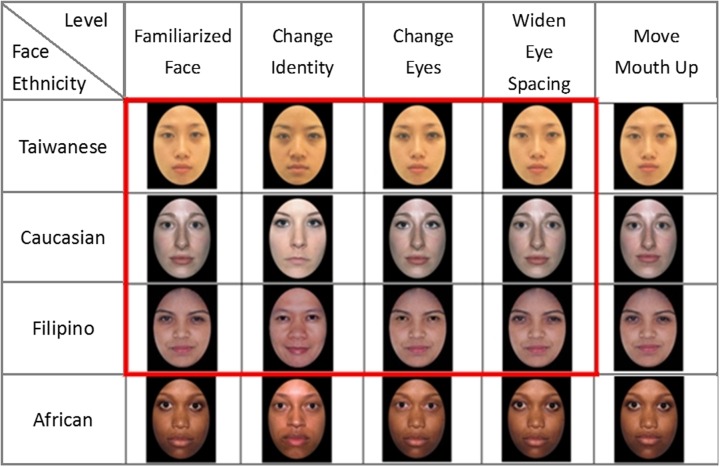
**The face stimuli used in the adult experiment and a subset of the stimuli used in the infant experiment (indicated with the red frame).** The stimuli contained female/male faces of four ethnic groups (Taiwanese, Caucasian, Filipino, and African) only female faces are shown here. Four levels of difficulty in stimulus discriminability are adopted: the “change identity,” “change eyes,” “widen eye spacing,” and “move mouth up” conditions.

In each race/ethnicity block, the sequential face discrimination task contained a single target face presented at the center and a pair of comparison faces, presented side by side with a distance of 15.5 cm in between. One image of the comparison faces was always identical to the target face, the other was a digitally altered one from the original target image to varied degrees according to the four different manipulations: (1) change identity : a different person’s face with the same race and gender, (2) change eyes: the same face with two eyes replaced, and (3) widen eye spacing: the same face with eyes spacing widen by 14 pixels (7 pixels each eye), (4) move mouth up: the same face with mouth moved up by 10 pixels. The locations of the new and the target face images were counterbalanced. **Figure 1** illustrates sample female face stimuli of four ethnic groups with the four different manipulations.

#### Design and Procedure

The ethnicity of the stimuli (Taiwanese, Caucasian, African, Filipino), four manipulations (change identity, change eyes, widen eye spacing, move mouth up), and the gender of stimuli (male or female) were the three independent within-subject factors. The dependent variables were accuracy and reaction time. The sequential two-alternative-forced-choice (2AFC) old/new face discrimination task was adopted, which was meant to be comparable with the familiarization /visual-paired-comparison procedures in the infant experiment. There were four race blocks (256 trials in total) and the test order was counter-balanced among participants. Each block included 64 randomized trials (4 different manipulations ^∗^ 2 genders ^∗^ 2 locations ^∗^ 4 repetitions). Each trial started with a white fixation cross for 1 s, followed by a target face for 1.2 s. After a 0.3 s blank, two comparison face images appeared, and the participant was asked to choose the image that was different from the target by key press (i.e., “left” or “right”). Here “different” means not exactly identical. The comparison faces remained on the screen until the participant made a response. A trial was terminated if the participant did not make a response within 5 s. No feedback was given in any trial. Prior to the start of the first experimental block, participants were given six practice trials, with feedback, of images of two additional Taiwanese female faces that were not included in the formal experiment.

### Experiment 2: The Infant Study

#### Participants

A total of 59 healthy full-term infants aged between 81 and 295 days were recruited from the Taichung Metropolitan areas. The parents joined the study via the advertisements posted in the university affiliated hospitals, and/or from the internet parenting community (i.e., BabyHome) forum. Informed parental consent was obtained before the experiment. All infants met the screening criteria of (1) normal delivery, and (2) born within ± 14 days of their due dates, (3) birth weight between 2550 and 4000 g, and (4) no history of blindness or health problems reported by their parents. In addition, the infant participants had no prior exposure to other-race individuals or Philippines by parent’s report. The 4-month-old group comprised 20 infants; among which, four infants were later excluded due to inability to complete the minimum number of 12 trials (*n* = 3) or side bias (*n* = 1). As a result, 16 infants (nine boys) were retained in the final sample with an average age of 120.1 days, ranging from 85 to 147 days. The 6-month-old group was composed of 20 healthy infants; one infant was excluded due to insufficient number of trials. Nineteen infants (10 boys) were retained in the final data set with an average age of 183.4 days, ranging from 167 to 199 days. The 9-month-old group comprised 19 infants; three infants’ data were later excluded due to an inability to complete the minimum number of 12 trials (2) and an experimental error (1). Thus, 16 infants (eight boys) retained in the final sample, the average age was 266.8 days, ranging from 233 to 295 days. After the experiment, parents received cash compensation and a baby toy as a token of appreciation for their participation.

#### Apparatus and Stimuli

Because infants have limited attention span, we chose a subset of face stimuli used in the adult’s study (see **Figure 1**). First of all, we dropped African faces to reduce the number of ethnic groups to three, Taiwanese, Caucasian, and Filipino. Secondly, we used only female faces for infants. Lastly, as informed by the adult’s performance (see Result), we adopted three manipulations, the “change identity,” “change eyes,” and “widen eye spacing” conditions, while dropped the “move mouth up” condition because it seemed too difficult even for the adults. The same computer and software were used to run the experiment. An online video-camera surveillance system was placed on the top of the monitor to show real-time images of infant participant’s eye movements and head movements. Another hidden webcam (Logitech Webcam C500), mounted below the monitor display, recorded the infant’s looking behavior throughout the entire experiment.

#### Design and Procedure

**FIGURE 2:**
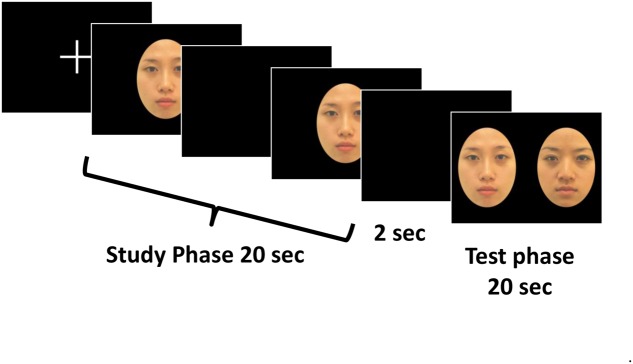
**Illustration of the familiarization/visual-paired comparison (VPC) procedure used is the infant experiment**.

To better assess infants capacity in discriminating own- vs. other-race faces, we adopted a within-subject design with Ethnicity (Taiwanese, Caucasian, and Filipino) and Manipulation (change identity, change eyes, widen eye spacing) as the two main stimulus variables. Each infant received three race/ethnicity blocks; the test order was counterbalanced among participants. Each block comprised 6 trials containing the “change identity,” “change eyes,” “widen eye spacing” conditions of two trials each (3 levels of manipulations ^∗^ 2 locations of the novel face image on the left or on the right), presented in random order. Thus, each infant received 18 trials in total. It is known that the familiarization/visual-paired-comparison (VPC) procedure (Fagan, 1970) has been widely used to study visual recognition memory and perceptual discriminability in infants. If an infant can discriminate between the familiarized stimulus and a novel one presented in the test phase, he/she will spend significantly longer time looking at the novel stimulus, known as a novelty preference. To better accommodate for the relatively large number of trials for young infants, as well as to be more comparable with the adult’s task, we adopted a variant of the familiarization/ VPC procedure that we had successfully tested in our previous studies to explore infant’s face perception (Hsu and Chien, 2011) and perceptual organization (Chien et al., 2011). The familiarization phase of the variant procedure contained two 10-s presentation of a single familiarizing face (a total of 20 s familiarization), while the test phase contained a 20-s presentation of two faces side-by-side, one was always the same familiar face image paired with a novel face image with three different manipulations. **Figure 2** illustrates the variant procedure used in the present infant study.

The experiment was conducted in a quiet room in the university. The infant was held by their parent(s), sitting in front of the monitor at a distance of about 50 cm. At the same time, the parents were asked to either close their eyes or look above the screen to avoid influencing their infants’ behaviors. An experimenter hidden from the infant’s view examined the infant’s looking through the video-camera surveillance monitor and controlled the pace of the experiment. As shown in **Figure 2**, each trial began with a white fixation cross on a black background, when the infant was judged to gaze at the monitor display; the experimenter initiated the familiarization phase. The familiarization face, accompanied by an alerting noise (about 1.2 s), was presented twice, 10 s for each, separated by a 1 s blank of complete blackness. The test phase began after a 2 s blank. Two comparison face images (i.e., the same familiar face image paired with a novel one with three different manipulations), accompanied by an attractive sound (about 1.2 s) to attract infant’s attention to the monitor, and were presented for 20 s. Meanwhile, the experimenter recorded the infant’s eye fixation and looking time spent on each of the face stimuli via a response coding program. The whole procedure was also videotaped through a hidden webcam and was later coded by another observer off-line with the same coding program.

As long as the infant participant completed at least two blocks out of three (or at least 12 trials in total), his/her datum would be retained in the final data set. The details of the final sample size for each race/ethnicity condition are as follows: 16 4-month-olds, 18 6-month-old, and 16 9-month-olds completed the Taiwanese condition; 14 4-month-olds, 19 6-month-old, and 16 9-month-olds completed the Caucasian condition; 15 4-month-olds, 18 6-month-old, and 16 9-month-olds completed the Filipino condition. Moreover for each trial, if the infant spent less than 2 s in the total looking time in either of the two familiarization episodes or in the 20-s test phase, his/her datum of that particular trial would be excluded. Each infant’s data were averaged by the two observer’s coding results. The averaged Pearson’s correlation strengths between the two observers were 0.896, 0.907, and 0.896 for 4-, 6-, and 9-month-old groups, respectively, indicating good inter-observer’s reliabilities.

## Results

### Experiment 1: Adult’s Data

The main purpose of the adult experiment was to ensure that the current stimulus manipulations with featural or configural changes provided appropriate levels of task difficulty and therefore a subset of the stimuli can be applied to the infant participants. Thus, here we only reported the analysis on accuracy^1^. A preliminary analysis revealed no effect on the participants’ sex, thus we dropped this factor and conducted a three-way repeated ANOVA with Ethnicity, Manipulations, and Stimulus gender as the within-subject factors. The results showed that the Ethnicity main effect was not significant (*p* = 0.288), the mean accuracies for Taiwanese (*M* = 79.84%, *SE* = 2.19%), Filipino (*M* = 78.54%, *SE* = 2.33%), Caucasian (*M* = 79.28%, *SE* = 2.35%), and African faces (*M* = 78.54%, *SE* = 2.29%) were about equal. The main effect of Stimulus gender was significant, *F*(1,21) = 12.58, *p* = 0.002, $\eta_{p}^{2}$ = 0.375; the mean accuracy for recognizing female faces (*M* = 82.10%, *SE* = 1.23%) was about 3% higher than that for male faces (*M* = 79.10%, *SE* = 1.53%).

As we had expected, the main effect of Manipulations was highly significant, *F*(3,63) = 130.82, *p* < 0.001, η^2^ = 0.862. Further analyses on pair-wised comparisons revealed that the differences in the mean accuracy between all six possible comparisons (i.e., change identity vs. change eyes, change identity vs. widen eye spacing, change identity vs. move mouth up, change eyes vs. widen eye spacing, change eyes vs. move mouth up, and widen eye spacing vs. move mouth up) were significant. The “change identity” condition had the highest mean accuracy (*M* = 98.71%, *SE* = 0.83%), indicating that it was fairly easy for the participants. The mean accuracy for the “change eyes” condition was about 90% (*M* = 90.71%, *SE* = 1.70%), showing that it was not as effortless as in the “change identity” condition. The mean accuracy for the “widen eye spacing” condition dropped to about 70% (*M* = 71.07%, *SE* = 1.06%). Lastly, the mean accuracy for the “move mouth up” condition has fallen below 60% (*M* = 56.55%, *SE* = 2.37%), suggesting that the last condition was rather difficult even for the adults. A mild ORE was revealed by a significant Ethnicity^∗^Manipulation interaction (*p* = 0.048). Further analyses on pair-wised comparisons in the “change identity” condition revealed that the mean accuracy for Taiwanese faces was significantly higher (*M* = 100%, *SE* = 0%) than African American faces (*M* = 97.86%, *SE* = 0.49%) and Filipino faces (*M* = 97.61%, *SE* = 0.68%).

**FIGURE 3:**
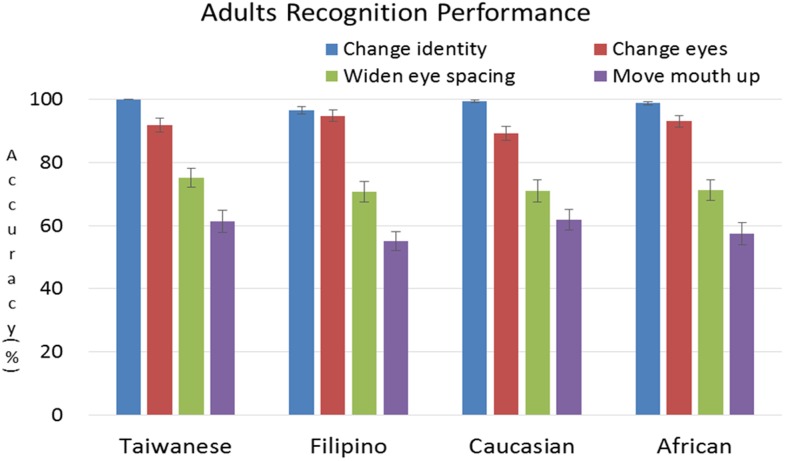
**The adults’ mean performances with the four different manipulations for each race/ethnicity.** The abscissa represents the four racial/ethnic groups (Taiwanese, Filipino, Caucasian, and African), while the ordinate depicts the mean accuracies for correctly discriminating the new image from the familiar ones. The color bars represent recognition accuracies with the four stimulus manipulations (blue: change identity; red: change eyes; green: widen eye spacing; purple: move mouth up). Error bars represent the standard errors (±SE) of the group means. The pattern of results clearly indicates that the adults’ response accuracies decrease as the manipulations change from identity, eyes only, eye spacing, to mouth spacing, and such tendency is present for each of the four race/ethnicity conditions.

**Figure 3** illustrates the adults’ performance with each manipulation for each race/ethnicity condition. The abscissa represents the four races/ethnicities while the ordinate depicts the participants’ mean accuracies with the colors representing the four different manipulations. As shown in **Figure 3**, the adults’ mean accuracy decreases as a function of stimulus manipulation in the following order “change identity” > “change eyes” > “widen eye spacing” > “move mouth up.” In other words, the adults found it easiest to detect a change in identity and hardest to detect a spacing difference near the mouth area, and this order of difficulty is present for each race/ethnicity condition.

### Experiment 2: Infants’ Data

In each trial, an infant’s fixation time during the familiarization phase and the test phase were coded by two observers. Each infant’s data were then averaged by the two observer’s coding results. To fully reveal whether an infant exhibited a novelty preference, we computed two kinds of indices of the looking data. First, the delta-looking time (in second) was indexed by subtracting the time spent looking at the familiar stimulus from the time spent looking at the novel stimulus. This index highlights the “net” difference in the fixation duration between the novel and the familiar face image (Sugita, 2008). In addition, a positive value indicates a preference for the novel image; a negative value for the familiar image; a value close to zero means no preference, which made the data pattern easier to understand at a glance. Second, to minimize the individual differences in the overall looking durations among infants and to adhere to the more traditional measure of novelty preferences, we also computed the index of “novelty preference” score. The novelty preference score is expressed in percentage and was computed as the time spent looking at the novel stimulus divided by the total looking time to stimuli. Thus, these data were analyzed by comparing the preference scores with the 50% chance level to determine if a significant novelty preference is present. Both indices can tell us whether a successful discrimination occurs for a particular stimulus condition.

#### Fixation Time in the Familiarization Phase

Preliminary analysis revealed no significant gender difference for the participants, thus the data were collapsed across the participant’s gender in subsequent analyses. As there were 6 trials in one race block, the familiarization face appeared six times (with a fixed presentation of 20 s each time). Thus, we added up the infants’ fixation durations during familiarization phase for the first three trials and the last three trials separately, and expected to see a decline in fixation duration from the first half to the second half of the six trials. This will be a good index showing infants were indeed well familiarized with the target face as the trials proceeded. A mixed three-way ANOVA on familiarization time (in seconds) was conducted with Ethnicity (Taiwanese, Caucasian, or Filipino), Order (the first three vs. the last three trials) as the within-subject factors and Age group (4, 6, or 9 months) as the between-subject factor. First of all, the Ethnicity main effect was not significant, meaning that infants spent about equal of time looking at the Taiwanese, Caucasian, and Filipino familiarizing faces. As expected, the *Order* main effect was significant, *F*(1,48) = 28.25, *p* < 0.001, η^2^ = 0.386, in which the accumulated fixation time for the first three trials (*M* = 29.35 s, *SE* = 1.19 s) was significantly greater than that of the last three trials (*M* = 25.53 s, *SE* = 1.20 s), indicating a decline in looking time as the trials progressed during an experimental block. The Age group main effect was also significant, *F*(2,48) = 3.69, *p* = 0.033, η^2^ = 0.141. *Post hoc* Scheffe tests revealed that the fixation time during familiarization phase for 4- and 6-month-old infants did not differ from one another, however, the fixation times for both 4-month-old (*M* = 29.76 s, *SE* = 1.97 s) and 6-month-old infants (*M* = 29.66 s, *SE* = 1.85 s) were marginally longer (*p* = 0.066, *p* = 0.070) than that for 9-month-old infants (*M* = 22.91 s, *SE* = 2.10 s). None of the two-way or three-way interactions were significant, either. In addition to the test order analysis, we also analyzed whether infants’ looking time may differ as a function of face race and difficulty levels. Again, as there were six trials in one race block with each difficulty level appearing twice in randomized order, we averaged the infants’ fixation time during familiarization for the two “change identity,” “change eyes,” and “widen eye spacing” trials separately. Therefore, we ran another mixed three-way ANOVA on familiarization time (in seconds) with Ethnicity, Manipulation (change identity, change eyes, widen eye spacing) as the within-subject factors and Age group as the between-subject factor. Again, the Age group main effect was significant. However, neither the Manipulation nor the Ethnicity main effect was significant. None of the two-way or three-way interactions were significant, either.

#### Delta Looking Time Analysis in the Test Phase

Again, preliminary analyses yielded no significant gender differences for the participants, thus the data were collapsed across gender in all subsequent analyses. We conducted a three-way mixed ANOVA on the delta-looking time (see above definition) with Ethnicity (Taiwanese, Caucasian, Filipino) and Manipulation (change identity, change eyes, widen eye spacing) as the within-subject factors while Age group (4-, 6-, 9-month-olds) as the between-subject factor. Results showed that both the Age group and Ethnicity main effects were not significant. The main effect of Manipulation was highly significant, *F*(2,74) = 9.46, *p* < 0.001, η^2^ = 0.204. Subsequent *post hoc* tests revealed that the mean delta looking time for the “change identity” condition (*M* = 1.95 s, *SE* = 0.35 s) was significantly higher than those for the “change eyes” (*M* = 0.72 s, *SE* = 0.37 s, *p* = 0.011) and the “widen eye spacing” conditions (*M* = -0.53 s, *SE* = 0.45 s, *p* < 0.001). No other two-way or three-way interaction terms reached statistical significance.

**FIGURE 4:**
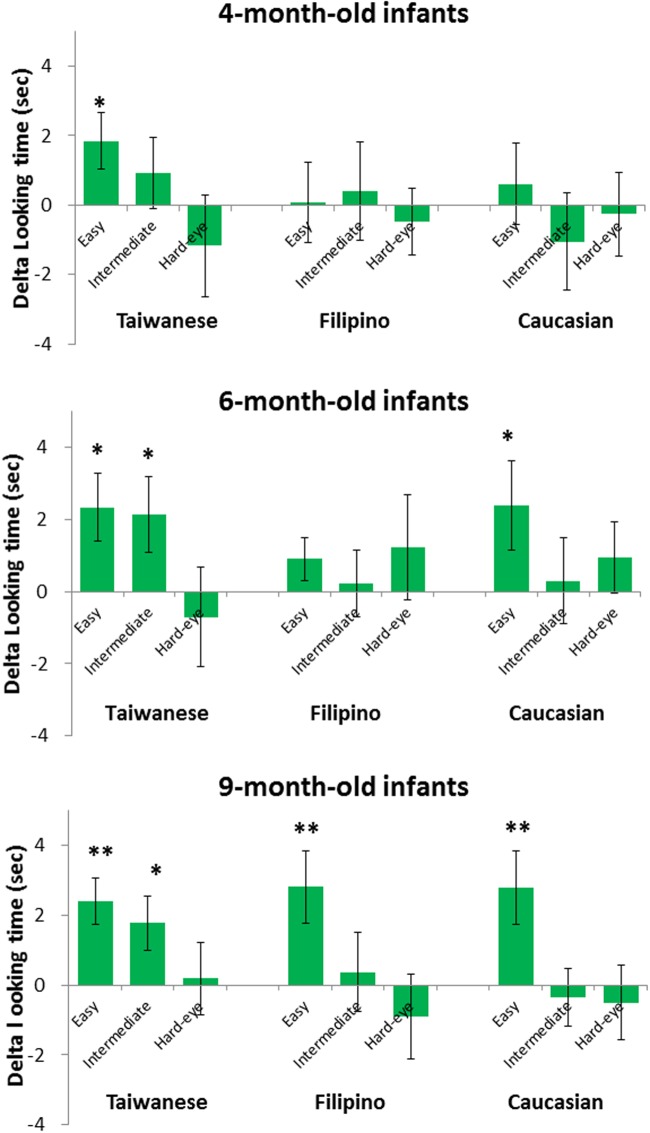
**The delta looking time results for the 4-month-old **(top graph)**, 6-month-old **(middle graph)**, and 9-month-old **(bottom graph)** infants.** The group mean delta looking times for each difficulty conditions in each ethnicity block are shown in the graph. The abscissa represents the “change identity”(easy), “change eyes”(intermediate), and “widen eye spacing”(hard-eye) conditions for the Taiwanese, Filipino, and Caucasian faces, while the ordinate depicts the group means of the delta looking time. The error bars represent the standard errors (±SE) of the group means. Level of significance: ^∗^*p* < 0.05; ^∗∗^*p* < 0.01; ^∗∗∗^*p* < 0.001.

One important goal of the present study was to examine whether infants exhibit differential developmental changes in discriminating own- vs. other-race faces. We addressed this by analyzing their performances at different types of stimulus manipulations. To better reveal these possibly subtle effects, we conducted separate sets of two-tailed *t*-tests for each manipulation conditions of each ethnicity/race within each age group. **Figure 4** illustrates the 4-month-old (top panel), 6-month-old (middle panel), and 9-month-old (bottom panel) infants’ mean delta-looking times as a function of the three manipulations in each ethnicity block. The abscissa represents the change identity; change eyes, and widen eye spacing conditions for the three ethnicity/races, while the ordinate depicts the group means of the delta-looking time. As shown in **Figure 4**, 4-month-old infants did not show any statistically significant positive delta-looking time except for the Taiwanese “change identity” condition, *M* = 1.81 s, *SE* = 0.76 s, *t*(15) = 2.38, *p* = 0.031. This indicates early ORA at 4 months. 6-month-old infants made noticeable progress; they passed the Taiwanese “change identity” condition, *M* = 2.39 s, *SE* = 0.93 s, *t*(17)^2^ = 2.58, *p* = 0.019, as well as the Taiwanese “change eyes” condition, *M* = 2.25 s, *SE* = 0.89 s, *t*(17) = 2.52, *p* = 0.022, indicating a refinement in the ability to processing own-race faces that they came to notice subtle differences in the eyes region. At the same time, 6-month-olds also passed the Caucasian “change identity” condition, *M* = 2.61 s, *SE* = 1.24 s, *t*(18) = 2.12, *p* = 0.049, but not the Filipino “change identity” condition. 9-month-olds maintained the discrimination for the Taiwanese “change identity,” *M* = 2.41 s, *SE* = 0.66 s, *t*(15) = 3.62, *p* = 0.002, and “change eyes” conditions, *M* = 1.77 s, *SE* = 0.77 s, *t*(15) = 2.31, *p* = 0.036. They also passed the Caucasian “change identity,” *M* = 2.79 s, *SE* = 1.05 s, *t*(15) = 2.67, *p* = 0.018, and the Filipino “change identity” conditions, *M* = 2.81 s, *SE* = 1.04 s, *t*(15) = 2.70, *p* = 0.017, but were unable to progress to the Caucasian or Philippine “change eyes” conditions.

#### Novelty Preference Score Analysis in the Test Phase

We also conducted a three-way mixed ANOVA on the preference score (in percentage) with Ethnicity and Manipulation as the within-subject factors and Age group as the between-subject factor. The results resembled the findings in the delta-looking time analyses. Likewise, the main effect of Manipulation was highly significant, *F*(2,74) = 8.00, *p* = 0.001, $\eta_{p}^{2}$ = 0.174. *Post hoc* comparisons revealed that the preference score for the “change identity” condition (*M* = 0.549, *SE* = 0.010) was marginally different (*p* = 0.057) from that for the “change eyes” (*M* = 0.525, *SE* = 0.010). These two conditions were significantly higher (*p* = 0.001, *p* = 0.030) than that for the “widen eye spacing” condition (*M* = 0.487, *SE* = 0.014). The main effects of Age group and Ethnicity were not significant; nor were any two-way or three-way interaction terms.

To further investigate novelty preferences within each age group, we conducted separate sets of two-tailed *t-*tests to determine whether the time spent looking at novel stimuli differed from the chance level of 50%, for each manipulation conditions of each ethnicity/race. **Table 1** illustrates the group mean novelty scores for each manipulation conditions of each face-ethnicity. As detailed in **Table 1**, 4-month-old infants showed a significant novelty preference only in the Taiwanese “change identity” condition, and not in any other ethnic conditions. Six-month-old infants demonstrated significant novelty preferences in the Taiwanese and Caucasian “change identity” conditions. Moreover, 6-month-olds made further progress in discriminating Taiwanese “change eyes” condition. Nine-month-olds demonstrated novelty preferences in the “change identity” conditions of all three ethnicities (Taiwanese, Caucasian, and Filipino). Their ability to discriminate own-race (Taiwanese) faces was more refined that they passed the “change eyes” condition, while their ability to process other-race (Caucasian or Filipino) faces stayed in the “change identity” conditions.

**Table 1:** The mean novelty preference scores for the three age groups.

	Novelty Preference Score^a^			
	
	*M* (%)	*SE* (%)	*t*^#^	*p*
**Four month-old infants**
Taiwanese face conditions				
Change identity	56.6	2.6	2.529	0.023^∗^
Change eyes	54.3	5.2	0.823	0.423
Widen eye spacing	47.8	5.8	-0.384	0.706
Caucasian face conditions				
Change identity	47.6	5.8	-0.421	0.681
Change eyes	46.3	4.2	-0.887	0.396
Widen eye spacing	50.5	3.3	0.143	0.888
Filipino face conditions				
Change identity	47.2	3.8	-0.741	0.472
Change eyes	51.8	5.5	0.331	0.746
Widen eye spacing	50.5	4.0	0.114	0.911
**Six month-old infants**
Taiwanese face conditions				
Change identity	56.3	2.7	2.328	0.033^∗^
Change eyes	55.4	2.4	2.268	0.037^∗^
Widen eye spacing	48.6	4.1	-0.343	0.736
Caucasian face conditions				
Change identity	56.7	2.9	2.333	0.032^∗^
Change eyes	50.1	2.7	0.030	0.976
Widen eye spacing	49.9	3.4	-0.039	0.969
Filipino face conditions				
Change identity	51.9	2.0	0.961	0.349
Change eyes	53.8	2.6	1.463	0.162
Widen eye spacing	51.7	3.1	0.556	0.585
**Nine month-old infants**
Taiwanese face conditions				
Change identity	55.7	2.0	2.899	0.012^∗^
Change eyes	55.3	2.3	2.320	0.035^∗^
Widen eye spacing	50.3	3.5	0.074	0.942
Caucasian face conditions				
Change identity	57.6	3.5	2.188	0.045^∗^
Change eyes	50.3	2.6	0.108	0.916
Widen eye spacing	47.1	3.3	-0.784	0.397
Filipino face conditions				
Change identity	58.4	3.3	2.521	0.024^∗^
Change eyes	49.7	2.9	-0.115	0.910
Widen eye spacing	47.2	4.4	-0.633	0.536


*^a^The novelty preference score is computed as the percentage of the looking time spent on the novel stimulus over the total looking time for both stimuli in the test phase of a particular trial. ^#^ The *t* statistic is computed as (*M* – 0.5)/*SE*, two-tailed *p*-value.*

*Level of significance: ^∗^*p* < 0.05; ^∗∗^*p* < 0.01; ^∗∗∗^*p* < 0.001.*

## General Discussion

Using a comprehensive set of face discrimination tasks, we asked whether infants aged between 4 and 9 months exhibit a general improvement in face processing as well as differential developmental changes in discriminating own- and other-race faces. The adult experiment was first conducted to ensure the appropriateness of stimulus manipulations and to guide the stimuli selection for the infant experiment. Several important findings were obtained. First, the adult data showed that the mean accuracies decreased as a function of stimulus manipulation in our expected order: change identity (∼98%) > change eyes (∼90%) > widen eye spacing (∼71%) > move mouth up (∼56%), indicating that the task difficulties created by the stimulus manipulations were valid. In fact, this order of performance was also consistent with our previous study testing 6- to 10-year-old healthy children with a similar set of stimuli and procedures (Chien et al., 2014). Moreover, a mild ORA was revealed in the Taiwanese “change identity” condition when compared with African American and Filipino faces. To rule out a possible confounding factor that the Taiwanese faces might have greater variation than the faces of the other race classes, we computed the physical similarity between the “familiarization” and “change identity” face of each race/ethnicity via an “Interactive Gabor-Jet Model Demo” website (Margalit, 2016, August 15)^3^ Based on the model’s outputs, which were distance scores (in arbitrary unit) between two faces, the visual (physical) similarity between the two Taiwanese faces was in fact greater (i.e., smaller distance scores) than the visual similarity between the Caucasian, African, and/or Filipino faces. In other words, the two Taiwanese faces were physically more similar rather than dissimilar. Thus, our result cannot be attributed to the concern that the selected Taiwanese faces were physically more distinguishable than faces of other race classes. Any subtle difference in the performance is likely to genuinely reflect the so-called “ORE” in processing own- vs. other-race faces with different proficiency.

Second, based on both the delta-looking time and novelty preference score analyses, 4-month-olds showed a significant novelty preference only in the Taiwanese “change identity” condition and not in any other ethnicity conditions, suggesting an early onset of ORA. This observation resonates well with several studies showing better sensitivity for own-race faces around 3 or 3.5 months of age (Sangrigoli and de Schonen, 2004; Hayden et al., 2007; Hsu and Chien, 2011; Tham et al., 2015). Third, 6-month-olds showed significant novelty preference in both the Taiwanese and Caucasian “change identity” conditions, an observation that actually agrees with the 6-month-olds’ data in Kelly et al. (2007b, 2009). Moreover, 6-month-olds further passed the Taiwanese “change eyes” condition, showing a refined ability to detecting a subtle featural change in the eyes region of own-race faces. The finding that the 6-month-olds detected a switch in the eyes is consistent with Geldart et al.’s (1999) study. They presented pairs of realistic, photographed female faces that were identical except for the size of the eyes; 5-month-old infants appeared to notice the difference and looked longer at the one with bigger eyes.

Last but not least, 9-month-old infants continued to exhibit novelty preferences in the “change identity” conditions of all three ethnicities, showing that infants at this age remain sensitive to differences not only in the own-race faces but also the other-race faces. Although this particular finding with 9-month-olds deviates from Kelly et al. (2007b, 2009), it agrees with a recent study with spatial cuing paradigm showing that 9-month-old Caucasian infants were able to discriminate faces regardless of race, in the focus of the attention bias (Markant et al., 2015). Nevertheless, it is worth noting that the 9-month-olds in our study continued to pass the Taiwanese “change eyes” condition but did not extend this ability to the Caucasian or Filipino “change eyes” conditions, suggesting that the refinement in face processing may be race-dependent. Furthermore, from 6 to 9 months, our oldest infants did not show any sign of novelty preference in the Taiwanese “widen eye spacing” condition, either. The 9-month-old infants’ failure in this condition suggests that the configural processing, specifically the second-order relational processing (Maurer et al., 2002) in detecting differences in spacing between two eyes, may be too difficult for infants at 9 months of age, at least for the cropped face stimuli used in the present study. Nevertheless, the absence of a second-order configural processing is consistent with the views that the development of configural processing may not begin until 4 years of age (McKone and Boyer, 2006) or even 5 or 6 years (Mondloch et al., 2006) and becomes more refined after that (c.f. Bhatt et al., 2005; Scott and Nelson, 2006).

### An Own-Race Advantage (for Female Faces) Is Readily Present at 3–4 Months

One key finding in the present study is the early emergence of an ORA; infants at 4 months already exhibited selective discriminability for own-race faces. Although this observation deviates from the studies favoring a non-selective sensitivity for own- and other-race faces at 3 months (Kelly et al., 2007b, 2009), it resonates with several studies of comparable age range. First of all, despite some differences in the manipulations of the test stimuli, the present study by and large replicated Hsu and Chien’s (2011), where they also tested 4-, 6-, and 9-month-old Taiwanese infants. The delta looking time data on Asian, Caucasian, and African faces revealed that an ORA for Asian faces was present at 4 months, and infants’ ability to recognize Caucasian and African faces emerged later around 6 months. Secondly, Sangrigoli and de Schonen (2004) showed that Caucasian infants, after habituation, could better recognize Caucasian faces than Asiatic ones, indicating 3-month-old Caucasian infants’ ability to process faces is already race-dependent (Experiment 1). If the infants were additionally familiarized with Asiatic faces, they then demonstrated successful recognition for both Caucasian and Asiatic faces (Experiment 2), indicating that the early ORE is highly plastic and can be learned with just a few exemplars.

Moreover, Hayden et al. (2007) used four face stimuli morphed from one Caucasian and one Asian female faces at different proportions (C100/A0, C70/A30, C30/A70, and C0/A100). They found 3.5-month-old infants were able to differentiate between the Caucasian-parent face (C100/A0) and a morphed face with 30% of the Asian component (C70/A30), but were unable to differentiate the same amount of physical differences in the Asian-parent condition (i.e., C0/A100 vs. C30/A70). This again demonstrated that 3.5-month-olds were already sensitive to “structural changes” in own-race faces, as if they exhibited a smaller discrimination threshold or just noticeable difference (JND) with the familiar race class.

Recently, Tham et al. (2015) proposed the idea that the timing of emergence of the ORE is dependent on face gender. They tested 3- to 4- and 8- to 9-month-old Caucasian infants with cropped faces of both genders showing in either frontal- or profile-view. They found an interaction with face gender: at 3–4 months, infants’ discrimination of Chinese, Malay, and Caucasian faces exhibited an ORA for female faces alone, whereas at 8–9 months, the ORA became general across gender. Note that the gender of the face stimuli in Sangrigoli and de Schonen (2004), Hayden et al. (2007), and the present study were all female. Taken together, it thus appeared that an ORA, at least for female faces, is readily present at 3–4 months.

The early ORA at about 3 or 4 months, found in the present study and those stated above, supports the notion that infants undergo a fast learning process for human faces in the first few months (Johnson and Morton, 1991; Elman et al., 1996; Turati et al., 2005; Chien, 2011; Otsuka, 2014; Johnson et al., 2015). Moreover, the fast learning process may manifest itself in the increasingly specialized brain circuitry responding to faces, as suggested by some recent developmental cognitive neuroscience studies that used positron emission tomography (PET) scans (Tzourio-Mazoyer et al., 2002), measured event-related potentials (ERPs) (de Haan et al., 2003), or near-infrared spectroscopic signals (NIRs) (Otsuka, 2014).

### Reconciling the Present Study with Studies Showing Broad Recognition at 3 Months

Up to date, it is generally agreed that the ORE develops within the first year, however, whether infants at 3–4 months already exhibit an own-race recognition advantage or a broad, non-selective recognition remains inconclusive. We will now address the inconsistent results between the present study and Kelly et al. (2007b, 2009). Several methodological differences in the experimental design, procedures, and the characteristics of the face stimuli between the two studies could have all contributed to these inconsistencies. For example, the factor of race/ethnicity was as a between-subject variable in Kelly et al. (2007b), Kelly et al. (2009) while it was a within-subject variable in the present study. For the testing procedures, Kelly et al. (2007b, 2009) used the habituation method to familiarize the infants and two 5-s test trials to obtain novelty preference, whereas we adopted a 20-s familiarization procedure and each test trial lasted for 20 s. Note that the duration of the total familiarization and the test trials can drastically alter the outcomes of infant’s looking preference in face discrimination task has duly pointed out (Fair et al., 2012).

Perhaps the most critical differences lie in the face stimuli. Tham et al. (2015, p. 132) suggested that two main differences in the stimuli, *gender* and *external facial cues*, could explain the inconsistencies among these studies. For instance, Sangrigoli and de Schonen (2004), Hayden et al. (2007) and the present study used cropped female faces while Kelly et al. (2007b, 2009) used both female and male full faces with external cues. Let us be specific about the possible role of external facial cues. Kelly et al. (2007b, 2009) adopted the frontal- and 3/4 views, full faces showing clear external cues such as hair, hairline, ear, and parts of the chin, whereas the present study as well as the aforementioned ones used frontal-view, oval-cropped faces without external cues such as hair, hairline, ear, and parts of the chin (Hayden et al., 2007; Hsu and Chien, 2011), or faces covered with shower cap (Sangrigoli and de Schonen, 2004). As such, the external facial information in Kelly et al. (2007b, 2009) may have provided additional visual cues permitting identification for young infants who might be more attracted to areas with high contrast and low spatial frequency. For example, the presence of hairlines highlights an edge signal of low-spatial frequency (i.e., global shape of the face) and high luminance contrast (i.e., light skin pit against dark hair yielding large difference in luminance), and these signals may better attract younger infants’ looking. Indeed, there has been evidence showing that very young infants tend to process the external features of faces more easily than their internal parts (Maurer and Salapatek, 1976; Pascalis et al., 1995; Turati et al., 2006) and shift to use more internal facial information instead from 5 to 9 months (Rose et al., 2008).

### Perceptual Learning: Own-Race Experience Facilitates the Specialization for Own-Race Faces

As summarized in **Table 2**, with continued exposure to own-race faces, the emergent ability among infants to discriminate among Taiwanese faces at 4 months progressed to the “change eyes” level at 6 and 9 months. Moreover, our infant participants became able to discriminate Caucasian and Filipino faces at 6 and 9 months, respectively, and their processing capacity stay at the “change identity” level rather than being diminished. This suggests that the lack of exposure to other-race/ethnicity faces does not lead to deterioration in the ability to discriminating among the other-race faces, at least for the cropped female face stimuli used in this study. Therefore, based on the current set of stimuli and data, our findings lend support for the perceptual learning view and regard the effect of own-race experience as *facilitative*, as opposed to *maintaining*, in the development of the visual discrimination among faces.

**Table 2:** The overall pattern of discriminability for own-race and other-race faces in 4-, 6-, and 9-month-old infants.

Age	Taiwanese			Caucasian			Filipino		
			
	Change identity	Change eyes	Change spacing	Change identity	Change eyes	Change spacing	Change identity	Change eyes	Change Spacing
4-month-old	^•^								
6-month-old	^•^	^•^		^•^					
9-month-old	^•^	^•^		^•^			^•^		


*The black circles represent the conditions in which infants show significant novelty preferences. Four-month-old infants can only discriminate the Taiwanese “change identity” face condition; 6-month-old infants can further discriminate the Taiwanese “change eyes” and Caucasian “change identity” conditions; 9-month-old infants can discriminate the “change identity” face conditions in all three ethnicities. At the same time, 9-month-old infants continue to pass the Taiwanese “change eyes” condition but cannot extend this capacity to the Caucasian or Filipino “change eyes” conditions.*

Moreover, we consider that the facilitative effect of own-race experiences entails *specialization* for frequently exposed face stimuli, Taiwanese faces in our case. Additionally, the early own-race experience somehow also facilitates the processing of infrequently exposed stimuli, Caucasian and Filipino faces in our case, perhaps owing to the shared inherent similarities in stimulus structure across own- and other-race human face classes (Dahl et al., 2014). For example, recent studies exploring infants’ spontaneous preference for own- vs. other-race faces suggested that the preference for own-race faces at 3 months shifts toward other-race faces between 6 and 9 months. Using pairs of African and Caucasian faces, Fassbender et al. (2015) tested 3-, 6-, and 9-month-old German Caucasian infants and found that the spontaneous own-race preference at 3 months reported in the literature tilts to a preference for other-race faces at 9 months passing through a null-preference at 6 months, a finding that replicated Liu et al. (2015)’s results with Chinese infants. Moreover, at all three ages, the infants’ fixation time toward Caucasian faces significantly reduces across the consecutive trial presentation. This can be explained in terms of more proficient own-race face processing at 3 months and beyond; infants need increasingly less time to form a mental representation of own-race faces, signaling an effect of “*specialization”* with more experienced own-race face stimuli.

Moreover, a recent computational study using a Bayesian model of visual-paired comparison task may shed some light on how other-race face recognition can also benefit from exposure to own-race faces. Balas (2012) explored how the acquisition of experience in face-space and the presence of race categories may affect recognition performance for own- and other-race faces. A training face set containing 90% of own-race faces and 10% of other-race exemplars was first conducted. The simulation results showed clear improvements in the recognition performance for both own- and other-race faces, with greater performance in the own-race face condition, as a function of the number of exemplars (*n* = 16, 32, or 64), analogs to the progression of age (Balas, 2012, p. 585, **Figure 4**). Moreover, the ORE was obtained consistently when racial categories were present, and was less evident when racial categories were absent at training. Most importantly, the author repeated the simulations with the pure own-race face training set (i.e., 100% own-race face and 0% other-race face exemplars) to see if the model would reproduce the same effects as seen with the 10% other-race face training set. Overall, the two batches of results exhibited good numerical agreements; a similar pattern of improvement in the recognition performance for both own- and other-race faces as a function of the number of exemplars was obtained with the pure own-race training condition.

In a nutshell, it appears that with enriched experience in own-race faces, infants become *specialize* in recognizing and discerning the subtle difference between faces of their own race, a specialty represented by improved recognition accuracy, shortened familiarization time, or enhanced performance on more difficult tasks (i.e., detecting a subtle change in the eyes). At the same time, with little to no experience in viewing other-race faces, the own-race experience somehow also facilitates the recognition of other-race faces, but to a lesser degree, a phenomenon reflected by a later emergent discriminability for other-race faces that stays at the baseline.

## Conclusion and Future Work

The ORE in face perception has been reliably demonstrated across ethnicities. Underpinned by interracial adoption studies as well as many others, ORE has been attributed to a lack of visual experience with other-race faces. Contentions arise as to its ontogeny and developmental pattern, with a special emphasis on the role of own-race experience. Deviated from the *perceptual narrowing* perspective, which would predict that infants do not show early selectivity and will “grow out of” the ability to discriminate between other-race faces, the present study provided an alternative possibility, the *perceptual learning* view, which regards the own-race experience as *facilitative*, in that it helps infants become *specialize* in recognizing and discerning the subtle difference in faces of their own race without deteriorating the ability to recognize other-race faces. In the present study, we demonstrated an early ORA at 4 months. Moreover, our findings suggest that age sharpens infants’ ability to process own-race faces as infants proceed from the “change identity” to “change eyes” and somehow makes other-race faces, including Caucasian, and Philippine faces, easier. Proposing a *perceptual leaning view* in the present study by no means undermines the validity of *perceptual narrowing* found in various aspects of language development, cross-modal perception, and the “other-species effect” in face perception. Here we simply provide an alternative possibility of *perceptual learning* view (at least for cropped female faces), manifested by improved sensitivity for frequently exposed stimuli, to capture the development of the ORE in the first year of life. Two limitations of the present study must be borne in mind: the physical similarity of the selected face stimuli of each race was not equated at the first place and an absence of convergent infants’ data from a different race/ethnicity. Therefore, an important avenue for future research is to use face stimuli allowing for better control of physical similarities, morphing faces for example (e.g., Hayden et al., 2007; Chen et al., 2016), or to select face pairs of about equal visual similarity for each race/ethnicity condition. Another important future research is to test the same comprehensive set of old/new face discrimination tasks with age-matched Caucasian infants and/or infants from other ethnic groups.

## Author Contributions

SHC and J-FW developed the study concept. All authors contributed to the study design. Testing and data collections were performed by J-FW, SHC, J-FW, and T-RH performed the data analysis and interpretation. SHC and T-RH drafted the manuscript. All authors approved the final version of the manuscript for submission.

## Conflict of Interest Statement

The authors declare that the research was conducted in the absence of any commercial or financial relationships that could be construed as a potential conflict of interest.

## Footnotes

1 We also analyzed the response time data. Likewise, the results showed that the response times increased as the stimulus difficulty increased, in the order of “change identity” being the easiest, followed by “change eyes” and “widen eye spacing,” and the most difficult one was the “move mouth up.” The pattern of results held true for each ethnic group. An own-race advantage was observed in the “change eyes” and “widen eye spacing” conditions in which the response time was lowest for the Taiwanese faces.

2 One infant in the 6-month-old group did not complete the Taiwanese face condition (only completed the Caucasian and Filipino conditions), thus the degree of freedom in the *t*-tests for the Taiwanese easy and intermediate conditions was 17 instead of 18.

3 http://geon.usc.edu/GJW/.
